# Recent Advances in Rechargeable Zn-Air Batteries

**DOI:** 10.3390/molecules29225313

**Published:** 2024-11-11

**Authors:** Hui Zhao

**Affiliations:** School of Materials Science and Engineering, Liaocheng University, Liaocheng 252000, China; zhaohui@lcu.edu.cn

**Keywords:** rechargeable Zn-air batteries, electrocatalysts, electrode engineering, electrolytes, battery configuration

## Abstract

Rechargeable Zn-air batteries are considered to be an effective energy storage device due to their high energy density, environmental friendliness, and long operating life. Further progress on rechargeable Zn-air batteries with high energy density/power density is greatly needed to satisfy the increasing energy conversion and storage demands. This review summarizes the strategies proposed so far to pursue high-efficiency Zn-air batteries, including the aspects of the electrocatalysts (from noble metals to non-noble metals), the electrode chemistry (from the oxygen evolution reaction to the organic oxidation reaction), electrode engineering (from powdery to free-standing), aqueous electrolytes (from alkaline to non-alkaline) and the battery configuration (from liquid to flexible). An essential evaluation of electrochemistry is highlighted to solve the challenges in boosting the efficiency of rechargeable metal-air batteries. In the end, the perspective on current challenges and future research directions to promote the industrial application of rechargeable Zn-air batteries is provided.

## 1. Introduction

For a long time, fossil fuels have supported the development of industrial civilization but also brought practical challenges, such as environmental pollution and climate change, that affect human survival and development. The energy production and consumption methods based on fossil fuels urgently need to be transformed. At the same time, clean energy power generation, such as wind and solar energy, is generally in an accelerated development stage and still faces significant challenges owing to its intrinsically intermittent nature and fluctuating output power. The rechargeable energy storage device can act as a reservoir to accommodate the energy and give a stable electrical output. Rechargeable metal air batteries (MABs) are a special type of fuel cell and one of the representatives of the new generation of green secondary batteries [1,2,3]. MABs leverage the advantages of fuel cells, using oxygen in the air as the active material of the positive electrode and metal zinc, aluminum, lithium, etc., as the active material of the negative electrode. Oxygen in the air can continuously reach the electrochemical reaction interface through gas diffusion electrodes and react with metal, zinc, or aluminum to release electricity. MABs have the advantages of low cost, environmental friendliness, high power density, and high energy density, thus showing great potential as efficient energy storage systems for large-scale industrial applications. However, one of the main barriers to further improvements in rechargeable MABs is the high overpotential on the air electrode.

The oxygen reduction reaction (ORR) occurring during the discharge process and the oxygen evolution reaction (OER) during the charging process at the air electrode both possess sluggish kinetics with complicated reaction mechanisms due to the involved 4e^−^ transfer process. It leads to a decrease in the overall energy efficiencies of MABs [4,5,6]. Noble metal-based materials, such as Pt-based materials for ORR and IrO_2_ and RuO_2_-based materials for OER, have been used as efficient electrocatalysts to boost the reaction rate [7,8]. However, the high cost, scarcity, and inferior bifunctional activity of noble metal-based catalysts are unfavorable for their industrial applications. Exploring non-noble metal-based bifunctional ORR and OER electrocatalysts is desperately needed to meet the performance requirements on the air electrode of MABs and thus enhance the battery performance. For instance, heteroatom-doped carbons, transition metal oxides, phosphides, and phosphates have been extensively investigated as promising ORR/OER catalysts [9,10,11]. The mechanism underlying the OER and ORR on electrocatalysts is important for the development of MABs [12,13]. Using a replaceable electrooxidation of organic compounds (EOO) with lower overpotential and more favorable kinetics compared with OER is an effective strategy to improve the overall energy efficiency of rechargeable MABs [12]. From the perspective of electrode design, the free-standing electrode can avoid the use of non-electrochemical active additives and expose more active sites. It can also promote the diffusion and transmission of ions and improve the conductivity of the electrode, which facilitates the enhancement of electrocatalytic activity. The solid-state flexible MABs show great application prospects as an efficient energy storage device for wearable electronic devices due to their safety and high energy density [13,14,15]. Although rechargeable MABs have made significant breakthroughs in the above research areas, there still exist many challenges to be solved.

Several critical reviews have reported a fine overview of electrocatalyst engineering, reaction mechanisms, electrolyte type manipulation, and battery configuration that affect the electrochemical performance of MABs. A series of MABs, including Zn-air, Na-air, and Li-air batteries, were studied regarding their reaction mechanisms, electrodes, and electrolytes, etc. [16,17,18]. Electrocatalysts, as the main component of electrode materials, have been summarized by many reviews. For instance, the design of precious-metal-free bifunctional OER/ORR electrocatalysts was summarized, and their applications in liquid and flexible MABs were analyzed [19,20]. Dendrite growth, corrosion, passivation, and hydrogen evolution reaction (HER) issues in metal anodes concerning the protection strategies in aqueous electrolytes were systematically discussed by Wang and co-workers [21]. Zhao et al. focused on the anti-CO_2_ strategies for MABs, with a major emphasis on CO_2_ adsorption/absorption material design, all-in-one anti-CO_2_ air cathode design, and new electrolyte development [22]. The various electrolyte systems, electrolyte modification, and seawater electrolytes were also discussed in MABs [23,24]. The flexible MABs concerning the flexible electrodes fabrication, the electrolyte exploitation and battery configurations were summarized and expected to use in the wearable MAB systems [25,26]. However, a comprehensive review of MAB systems from multiple angles, including key components of MABs, the structure-performance relationships, the critical evaluation of the electrochemistry, the latest promising applications, and the challenges and perspectives, is still very scarce. Among the various metal anodes investigated, Zn-air batteries (ZABs) attract the most interest in practical applications due to their high theoretical energy density (1086 Wh kg^−1^), low cost, and high safety.

**Scheme 1:**
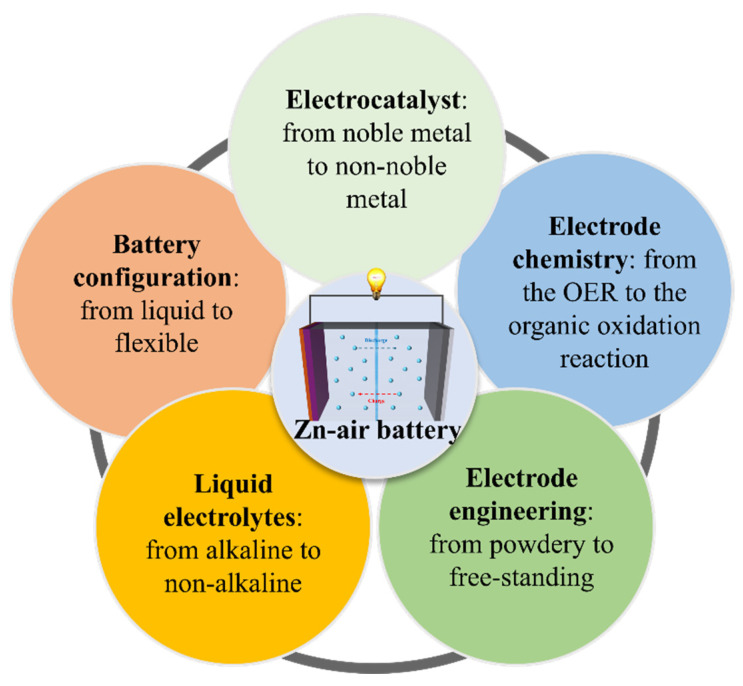
Illustration of the Zn-air batteries from five aspects, including the electrocatalyst, the electrode chemistry, the electrode engineering, the electrolyte, and the battery configuration.

In this review, we focus on the recent advances in rechargeable ZABs. We expect to highlight the ZAB systems from five aspects, as illustrated in Scheme 1, including the electrocatalyst, the electrode chemistry, the electrode engineering, the electrolyte, and the battery configuration. We first discussed the noble metal-based and non-noble metal-based electrocatalysts in improving the electrocatalytic performance. This is followed by a discussion on the electrode chemistry in ZABs. Then, we will give an overview of electrode engineering and highlight the current developments of the electrocatalytic organic oxidation reaction. After that, the aqueous and non-aqueous electrolytes were summarized. Subsequently, the liquid and flexible battery configurations are briefly introduced. Last, the current challenges and perspectives in rechargeable ZABs are provided.

## 2. Electrocatalyst: From Noble Metal to Non-Noble Metal

### 2.1. Noble Metal-Based Electrocatalysts

Noble metal-based electrocatalysts were the most used catalysts for the rechargeable ZABs due to their robust performances. Recently, diverse noble metal-based catalysts have been reported such as noble metal/noble metal alloys, noble metal/non-noble metal alloys and noble metal/non-metal composites [27,28,29,30].

**Figure 1:**
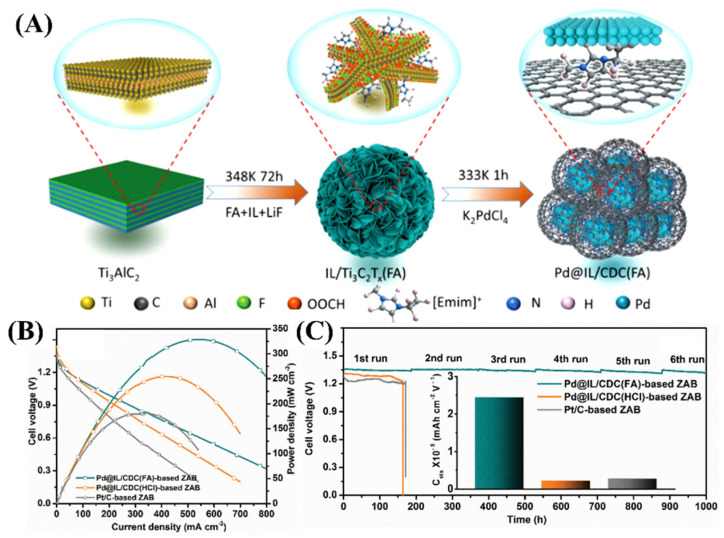
(**A**) Illustrating the preparation of surface-modified IL/Ti_3_C_2_T_x_ (FA) and one-step synthesis of Pd@IL/CDC(FA). (**B**) Discharge polarization and corresponding power density curves of the ZABs. (**C**) Discharge stability of the Zn-air batteries at a current density of 10 mA cm^−2^, inset: discharge stabilities of the Zn-air batteries. Reprinted with permission from [36], copyright, Wiley VCH.

The Pd_x_Au_y_ alloy nanoparticles were prepared via a polymerization-pyrolysis method [31]. It found that Pd-rich alloys possessed higher ORR performances than Au-rich alloys due to their optimal adsorption of *OOH. The Zn-air batteries (ZABs) with a Pd_55_Au_45_ alloy cathode exhibited a peak power density and specific capacity of 237.7 mW cm^−2^ and 821.4 mA h g^−1^, respectively, and 2000-h stability. The construction of metal-support interaction can alleviate the deactivation and degradation of noble metal electrocatalysts [32,33,34,35]. Carbon-based materials are commonly used to support and prevent metal dissolution and aggregation. The bilayer carbide-derived carbon (CDC)-encapsulated Pd nanocatalysts Pd@IL/CDC(FA) with a metal loading of 69.7% were prepared via the reduction encapsulation of ionic liquid/Ti_3_C_2_T_x_ (formic acid) (Figure 1A) [36]. Benefiting from both the nanoconfinement effects and the electronic effects, the catalyst exhibited high activity and good durability. As a result, Pd@IL/CDC(FA)-based ZAB delivered the specific capacity of 812.6 mAh g_zn_^−1^, the peak power density of 328.7 mW cm^−2^, the energy density of 1093.7 Wh kg_zn_^−1^ and the discharge stability for 1000 h (Figure 1B,C). Furthermore, single-atom noble metal catalysts with high atom utilization have been considered to improve electrochemical performances by lowering the energy barriers for the ORR/OER reactions [37,38,39].

Noble metal/non-noble metal alloys can also optimize the electronic structure and coordination environment compared to single noble metals. The nanoflower (NFs)-like carbon-encapsulated FeNiPt nanoalloy catalyst (FeNiPt@C NFs) was synthesized by carbonizing a FeNiPt-MOF precursor and a subsequent acid impregnation [40]. Due to the synergistic interaction between the phase-segregated FeNiPt active site and N-doped carbon-encapsulated metal alloy structure, the catalyst possessed high activity towards both ORR and OER in alkaline electrolytes. The catalyst-assembled ZABs showed a power density of 168 mW cm^−2^ and a specific capacity of 851.66 A h kg_zn_^−1^.

### 2.2. Non-Noble Metal Electrocatalysts

Although noble metal electrocatalysts exhibit superior catalytic performance, they exhibit various shortcomings, such as unifunctional activity, undesired stability, resource scarcity, and high cost, which greatly hinder their widespread applications in ZABs. In this regard, extensive efforts have been made to explore low-cost, highly efficient, and environmentally friendly non-noble metal-based electrocatalysts. Thus far, transition metals such as Co, Fe, Ni, and Mn, non-metals, such as carbonaceous materials, and their composites have been applied to be efficient electrocatalysts in ZABs [41,42].

The hybridization strategy of transition metals and porous carbon materials has been proposed for synergistic integration to achieve bifunctionality and facilitate mass transfer [43]. The cobalt nitride nanoparticles@cobalt oxynitride deposited on porous carbon aerogel (Co_4_N@CoON/PCGN) was prepared by a hydrothermal reaction and an in-situ nitridation process [44]. The theoretical calculations showed that the presence of a CoO layer covering Co_4_N can tune the adsorption energies of the OER reaction intermediates, while the Co-N_x_-C sites contribute to the high ORR activity. Owing to the strong coupling between CoO_x_N_y_ and conductive carbons, the catalyst exhibited high bifunctional activities towards both OER and ORR in alkaline electrolytes. When integrated into the ZABs, the catalyst demonstrated a voltage gap of 0.65 V and a battery lifetime over 1350 h at 5 mA cm^−2^. The introduction of heteroatoms such as N, S, P, and B into carbons can modulate their charge density and structure. The appropriate content can optimize the active sites and electronic structure of carbon materials, thus improving the catalytic activity [45]. Additionally, combining two metals in a hybrid catalyst can promote a synergistic effect for both ORR and OER [46,47]. For example, the catalyst based on an Ag and Co_3_O_4_ hybrid can be used as a highly efficient bifunctional catalyst for alkaline air electrodes [48].

**Figure 2:**
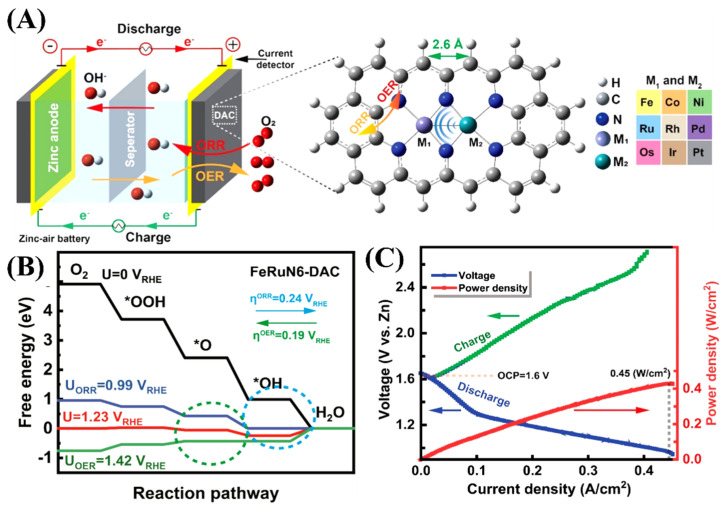
(**A**) The schematic of Zinc-air battery with M_1_M_2_N_6_-DAC. The metals are anchored on moiety sides. (**B**) The Gibbs free energy profiles of FeRuN_6_-DAC with the ORR and OER overpotentials of 0.24 V_RHE_ and 0.19 V_RHE_. * denotes a site on the surface. (**C**) Polarization curve and power density plots of an assembled Zinc-air battery using FeRuN_6_-DAC in place of the cathode for discharge and charge processes. This indicates an open circuit potential (OCP) of around 1.6 V. Reprinted with permission from [54], copyright, Elsevier.

Recently, transition metal single-atom catalysts have aroused much attention. Metal single atoms can pair with metal-based nanoparticles or another metal single atom to synergistically promote catalytic activity [49,50,51]. Fe single atoms/Fe_3_C nanoparticles @ S, N co-doped carbonaceous nano springs (FeNS/Fe_3_C@CNS) were synthesized by anchoring Fe species and thiophene onto the curved surface of twisted polypyrrole and a subsequent pyrolyzing process [52]. The presence of both Fe single atoms and Fe_3_C nanoparticles can act as active sites. The high curvature of the twisted surface and the presence of S could lower the energy barrier for ORR, thus improving the catalytic activity. When used FeNS/Fe_3_C@CNS as the air cathode to assemble flexible ZABs, it can achieve the peak power density and capacity of 193 mWcm^−2^ and 792 mA h g^−1^, respectively, and a high stability during at least 120 h. For the dual metal atom catalysts, one metal atom often acts as the active site, and another metal serves as the counterpart site, which can achieve the regulation of the spin state, d orbital electron distribution, and electronic structure of the metal sites [53]. For instance, FeRuN_6_ dual atom catalysts exhibited the optimal catalytic performance towards both ORR and OER compared with other M_1_M_2_N_6_ dual atom catalysts (M_1_ and M_2_: Fe, Co, Ni, Ru, Rh, Pd, Os, Ir or Pt) (Figure 2A) [54]. The theoretical calculation results showed that the ORR potential determining step is the proton transfer to OH* while that of the OER is the deprotonation of OH* (Figure 2B). Fe acted as the active site for both ORR and OER, and the interaction between Fe and Ru increased the spin state of Fe sites. When FeRuN_6_ dual atom catalysts were applied as the air cathode of a ZAB, it offered a maximum power density of 0.45 W cm^−2^ at 0.44 A cm^−2^ and good stability for 120 cycles (Figure 2C).

## 3. Electrode Chemistry: From the Oxygen Evolution Reaction to the Organic Oxidation Reaction

Although remarkable progress has been made in the development of bifunctional oxygen electrocatalysts, the performance of ZABs is still unsatisfactory. Recently, some small organic molecules (hydrazine, urea and glucose, etc.) oxidation reactions have been demonstrated to possess low theoretical thermodynamic potential, which are thermodynamically more favorable than OER in the alkaline electrolyte [55,56,57]. Therefore, replacing the OER with the organic oxidation reactions could decrease the charging voltage and increase the overall energy conversion efficiency of ZABs.

### 3.1. Urea Oxidation Reaction

Urea oxidation reaction (UOR) possesses a theoretical thermodynamic potential of 0.37 V vs. RHE and can reduce the pollution of water resources by urea [58]. By replacing the OER with UOR, the charging cycle in the UOR-assisted ZABs can be performed more facilely.

**Figure 3:**
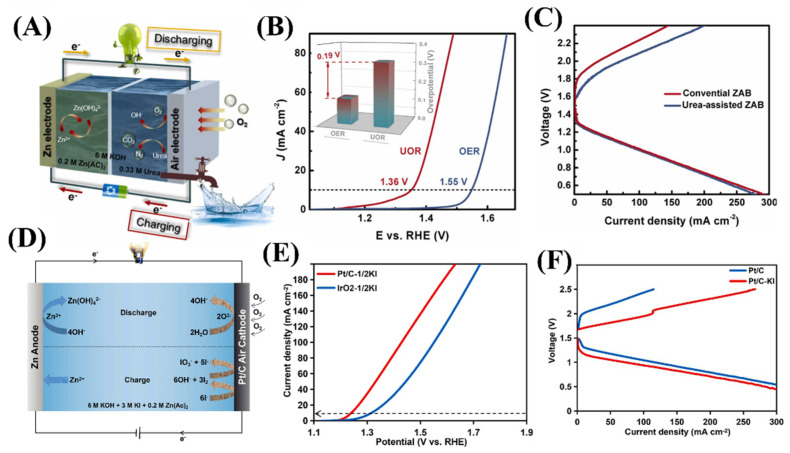
(**A**) Schematic illustration of the charging and discharging process for urea-assisted rechargeable ZABs. (**B**) LSV curves of CoNi@NCNTs −LDH/CC for UOR and OER. (**C**) Charging/discharging polarization curves for conventional and urea-assisted ZABs. Reprinted with permission from [59], copyright, Elsevier. (**D**) Schematic of the reactions within ZABs with KI additive during the charging and discharge process. (**E**) LSV curves of the Pt/C and IrO_2_ catalysts in 1 M KOH+ 1/2 M KI for IOR. (**F**) Charge and discharge polarization profiles of the Pt/C catalyst-based ZABs with and without KI. Reprinted with permission from [64], copyright, American Chemical Society.

CoNi@N-doped carbon nanotubes-layered double hydroxide/carbon cloth (CoNi@NCNTs-LDH/CC) composite materials were synthesized via a hydrothermal method using CoNi(CO_3_)_0.5_OH/CC as the precursor and a subsequent calcination and electrodeposition process [59] (Figure 3A). The spatially separated N-doped carbon nanotube and layered double hydroxide act as the active sites for ORR and UOR, respectively. The potential to drive 10 mA cm^−2^ for UOR was lower compared to OER (Figure 3B). The urea-assisted rechargeable ZAB based on the composite catalyst exhibited a reduced charging voltage while maintaining a superior discharging property, thus obtaining a higher energy conversion efficiency of 74.6% than that of ORR and OER-coupled ZAB (69.2%) (Figure 3C). Several catalysts such as Ni single atoms anchored on N-doped carbon nanosheets, LaNiO_3_ perovskite, and Co/CoSe_2_ heterojunctions have been reported to show bifunctional performance towards both ORR and UOR, thus improving the efficiency of ZABs [60,61,62].

### 3.2. Other Reactions

Electrocatalytic glucose oxidation reaction (GOR), due to its relatively low thermodynamic potential and abundant biomass source of glucose in nature, is a promising alternative to OER reaction in metal air batteries [63]. Recently, it was reported that by adding a redox radical 2,2,6,6-tetramethylpiperidinyloxyl and glucose into the electrolyte, the charging potential of a ZAB can be reduced to 1.6 V with an energy efficiency of 70% [64]. During charging, the 2,2,6,6-tetramethylpiperidinyloxyl oxidation can catalyze glucose oxidation, which restrains the dendrite growth of the Zn anode and allows the ZABs to exhibit long-term stability over 400 h. The I^−^ oxidation reaction (IOR) with lower oxidation potential and faster kinetics than OER has been explored to replace the OER and improve the efficiency of ZABs (Figure 3D). The commercial Pt/C catalyst shows remarkable IOR activity in alkaline electrolytes [65] (Figure 3E). The results showed the I^−^ is first electrochemically oxidized and then chemical disproportion to I^−^ and IO_3_^−^ in the alkaline media. The IOR-assisted ZABs achieved a low charging potential of 1.68 V, a high energy efficiency of 76.5% at 5 mA cm^−2,^ and a long cycle life of over 80 h (Figure 3F).

## 4. Electrode Engineering: From Powdery to Free-Standing

The commonly used air-cathode catalyst is made of powdery materials. However, the mass and ion transfer process during the electrochemical reactions may be hindered due to their low surface area, relatively poor porosity, and blocking of active sites. A binder such as Nafion solution is required to support the powdery catalysts on substrates, which may increase the resistance of the catalysts and block the active sites, thus leading to high cost and inferior catalytic performance. Free-standing porous electrodes with high surface area and 3D porous channels can circumvent the above issues. The free-standing electrode can be directly used as the air cathode in MABs without the binder, thus reducing the cost and improving the catalytic performance [66,67,68,69]. Moreover, the construction of free-standing electrodes facilitates the fabrication of solid-state MABs.

The catalysts can be grown on the conductive substrate via electrodeposition or chemical method to construct a free-standing electrode. Substrates, including metal foam, metal foil, carbon paper, and carbon cloth, have been commonly used in MABs. The in-situ coupling of Ni/Fe-NC and N-doped carbon nanofibers (NCF) on carbon cloth (Ni/Fe-NC/NCF/CC) was obtained by using a MOF as the precursor [70]. The catalyst possessed a 3D interconnected structure, thus facilitating the presence of abundant active sites and a fast mass transfer process. The ZAB with Ni/Fe-NC/NCF/CC air cathode exhibited a power density of 162.0 mW cm^−2^, a specific capacity of 770.5 mAh g_zn_^−1,^ and good durability for 2150 cycles.

**Figure 4:**
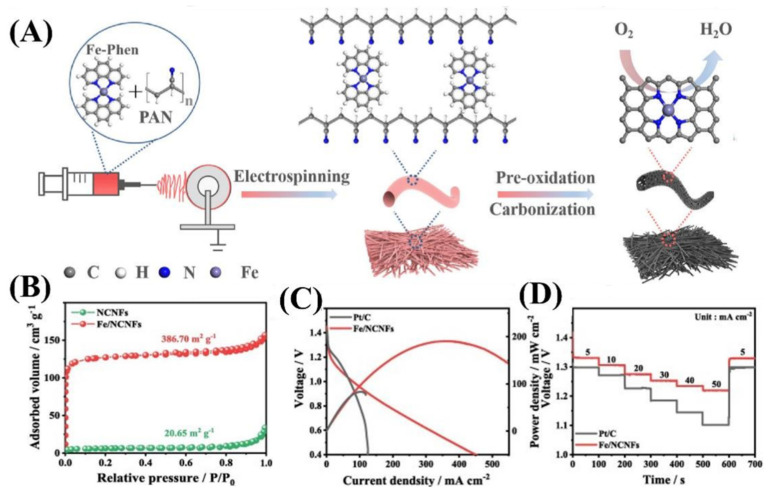
(**A**) Schematic of the preparation procedures of Fe/NCNFs. (**B**) N_2_ adsorption and desorption isotherms of Fe/NCNFs and NCNFs. (**C**) Discharge polarization curves and corresponding power density plots. (**D**) Rate performances of Zn−air batteries with the Fe/NCNFs and Pt/C electrocatalysts. Reprinted with permission from [72], copyright, Elsevier.

Moreover, the catalyst can be directly used as the electrode in the absence of substrate [71]. For instance, Fe/N co-doped carbon nanofiber membranes (Fe/NCNFs) with Fe-N_4_ active sites were prepared via electrospinning and pyrolysis method (Figure 4A) [72]. The catalyst exhibited a hierarchical micro/mesoporous structure and atomically dispersed Fe atoms, which facilitate the optimal exposure of active sites and the rapid diffusion of reactants (Figure 4B). Benefiting from the 3D porous structure and the abundant Fe-N_4_ active sites, the Fe/NCNFs-assembled liquid ZABs demonstrated a maximum power density of 190 mW cm^−2^, a specific capacity of 791 mAh g^−1^ and good durability for 2000 cycles (Figure 4C,D). The solid-state batteries with Fe/NCNF as the electrode also showed a high performance.

As stated above, most reported electrocatalysts are usually prepared in advance as suspensions and coated onto current collectors such as glassy carbon, which require the addition of binders to improve stability. In this case, the preparation of catalyst films is time-consuming and laborious, and the addition of polymer binders can affect conductivity and catalytic activity. Meanwhile, the process of oxygen evolution can cause the catalyst to peel off from the electrode, thereby reducing catalytic activity and stability. The construction of free-standing electrodes is favorable for the improvement of catalytic activity and battery performance. From an economic perspective, the bulk preparation of large-scale free-standing electrodes is highly desirable.

## 5. Liquid Electrolytes: From Alkaline to Non-Alkaline

The common electrolyte system for ZABs is alkaline electrolytes such as aqueous KOH and NaOH solutions due to the favorable ORR/OER reaction kinetics and their high solubility for Zn salts. However, alkaline ZABs undergo a loss of energy efficiency after certain charge-discharge cycles, which is mainly due to the dendrite growth, hydrogen evolution, electrode corrosion, and passivation of the Zn anode. The carbonate precipitates may exist owing to the presence of air contaminants such as CO_2_ [73,74,75]. Therefore, neutral/near-neutral electrolytes have recently been explored to alleviate the above problems. However, neutral electrolytes often possess low OH^−^ concentrations and poor ion conductivities, thus leading to sluggish ORR/OER reaction kinetics. Herein, exploring highly efficient air cathodes for neutral ZABs is indispensable. Currently, neutral electrolytes, including inorganic salts, organic salts, and quasi-solid-state electrolytes, have mainly been reported for ZABs.

### 5.1. Inorganic Salt Electrolytes

**Figure 5:**
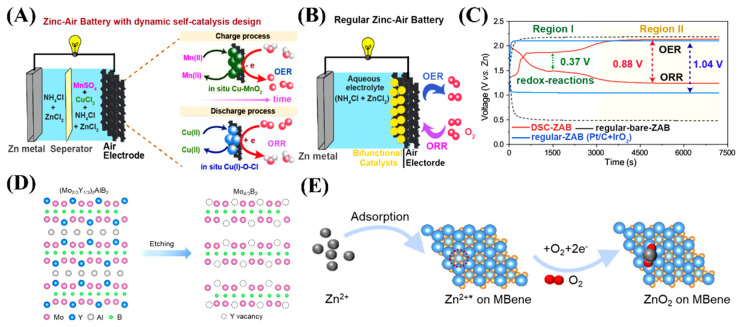
(**A**) The configuration of the rechargeable near neutral−ZAB with a dynamic self-catalysis design that involves a separator (anion-exchange membrane) and decoupled electrolytes. (**B**) A typical configuration of a regular rechargeable near neutral-ZAB with bifunctional catalysts preloaded on the air electrode. (**C**) The galvanostatic discharge–charge profiles of dynamic self-catalysis−ZAB, regular−ZAB with Pt/C + IrO_2_ catalysts (regular−ZAB(Pt/C+IrO_2_)), and regular−ZAB without any catalysts (regular-bare−ZAB) at a current density of 0.5 mA cm^−2^. Reprinted with permission from [78], copyright, The Royal Society of Chemistry. (**D**) The schematic atomic structure of (Mo_2/3_Y_1/3_)_2_AlB_2_ before etching (**left**) and after etching (**right**). (**E**) Schematic illustration of the ORR pathways for ZnO_2_ generation on the Mo_4/3_B_2−x_T_z_ MBene surface. * denotes a site on the surface. Reprinted with permission from [86], copyright, The Royal Society of Chemistry.

Inorganic salt electrolytes such as NH_4_Cl can alleviate Zn dendrites and avoid carbonation, thereby endowing ZABs with better battery stability. The chloride electrolytes can improve the cycling stability of ZABs. However, inert products such as ZnCl_2_·2NH_3_ will be formed to decrease the energy density of the battery [76]. The possible chlorine evolution and the poison of the catalyst cannot be ignored due to the presence of Cl^−^ ions, which have a negative significance on the O_2_ electrocatalysis and further deteriorate the battery performance [77]. Recently, the chloride electrolyte was reported to be essential for the in-situ formation of electrocatalysts. The dynamic self-catalysis generates the OER-active Cu-MnO_2_ and ORR-active Cu(I)-O-Cl electrocatalysts in situ from the NH_4_Cl-ZnCl_2_ electrolyte, which is triggered by Mn(II)/MnO_2_ and Cu(II)/Cu(I) redox reactions during the battery charge and discharge processes, respectively [78] (Figure 5A,B). The voltage gap of the redox reactions of the dynamic self-catalysis ZAB was 0.37 V, and the energy density was about 69% at 0.5 mA cm^−2^ (Figure 5C). The near-neutral ZABs with self-catalysis can offer a cycling stability of 1800 h, an areal capacity of 10 mAh cm^−2^, and a record-high energy efficiency of 69%. The in situ generating process did not efficiently occur in the sulfate-based electrolyte, indicating the importance of chloride electrolyte.

In addition to the chloride electrolyte, PBS buffer has also been reported as the neutral electrolyte [79]. For instance, the atomic Fe-N_4_ sites and Fe nanoclusters on N-doped porous carbon (Fe_SA+NC_@NMPC) were synthesized via self-assembly and subsequent pyrolysis methods [80]. Benefiting from the synergistic effect between atomic Fe-N_4_ sites and Fe nanoclusters, the Fe_SA+NC_@NMPC-based neutral ZABs in 0.1 M PBS achieved the specific capacity of 799.3 mAh g^−1^ and the cycling stability at 5 mA cm^−2^ for 100 h.

### 5.2. Organic Salt Electrolytes

Introducing suitable amount of organic solvent could minimize the HER as much as possible and promote battery performance [81,82,83]. The organic salt electrolytes can affect the reaction kinetics of ORR and OER and coordinate with Zn^2+^ to regulate the discharge product. It has been reported that the near-neutral ZAB with 1 M zinc trifluoromethanesulfonate (Zn(OTf)_2_) electrolyte can achieve the stability of 1600 h at 0.1 mA cm^−2^ [84].

The mesoporous carbon was synthesized by using a solvent evaporation-induced self-assembly approach [85]. The as-synthesized carbons possessed both micropores and mesopores and a specific surface area of 1081 m^2^ g^−1^. The resultant near-neutral ZABs in 1 M Zn(OTf)_2_ with the carbon cathodes exhibited a polarization of below 0.62 V, an energy efficiency of over 73%, and a cycle life of over 238 h at 1 mA cm^−2^. The results showed that the discharge product was evenly distributed on the surface and disappeared after charge in the Zn(OTf)_2_ electrolyte, indicating good electrochemical reversibility on the carbon cathode. In another example, Mo_4/3_B_2−x_Tz MBene with ordered Y vacancies was synthesized via an exothermal reaction between the HF and the 3D (Mo_2/3_Y_1/3_)_2_AlB_2_ (MAB) phase [86] (Figure 5D). The Mo_4/3_B_2−x_Tz MBene exhibited high O_2_-adsorbability and air stock capacity. The catalyst-based coin cell near-neutral ZABs with Zn(OTf)_2_ electrolyte presented a high reversibility for 380 h at 2 mA cm^−2^ with a 5 h cycling duration. The results showed that the presence of Y vacancies was favorable for reversible 2e^−^ ORR and OER processes, thus promoting the sluggish kinetics of ZnO_2_ chemistry (Figure 5E). The formation of discharge product ZnO_2_ occurs during the ORR process and then decomposes during the OER process.

## 6. Battery Configuration: From Liquid to Flexible

In recent years, significant development has been made in the field of wearable and portable electronic products. To power wearable electronic devices, there is an urgent need for flexible energy storage devices with high safety, high energy density, long cycle life, and bending tolerance. Flexible ZABs have attracted a lot of attention due to their high specific energy density, excellent stability, and high safety [87,88,89,90]. The construction of flexible ZABs requires flexible free-standing electrodes and quasi-solid-state/solid-state electrolytes to ensure their safety and mechanical robustness.

### 6.1. Quasi-Solid-State/Solid-State Electrolytes

The currently used quasi-solid-state/solid-state electrolytes for flexible ZABs are gel polymer electrolytes (GPE) such as polyvinyl alcohol (PVA), polyacrylic acid (PAA), poly(ethylene oxide) (PEO), and polyacrylamide (PAM) [91,92,93,94].

PVA-based electrolytes such as PVA-KOH-based electrolytes have been widely used due to their high hydrophilicity, easy preparation, and good chemical stability. However, the pure PVA electrolyte possesses low ion conductivity, weak electrolyte uptake capability, and inferior mechanical properties. Thus, certain electrolyte additives, including PEO, graphene oxide (GO), and tetraethylammonium hydroxide (TEAOH), into PVA-based electrolytes have been reported to solve the above issues [95,96,97]. As reported, a physically cross-linked double network hydrogel consisting of a strong agar as the first network and a flexible PVA as the second network was prepared as GPEs [98]. The introduction of GO nanosheet additive can act as a macromolecule adsorption platform to enhance both the ionic conduction rates and the mechanical properties of the gel electrolyte. The results showed that the alkaline GPE owns high ionic conductivity (75 mS cm^−1^) and good water retention, leading to a power density of 123.7 mW cm^−2^ of the assembled alkaline flexible ZAB. The neutral GPE possessed a good water retention of 76.3% after 24 h, leading to a long discharge time and good cycle life of more than 60 h of the assembled neutral flexible ZAB.

PAA-based GPE has emerged as a promising alternative to PVA-based GPE due to its high ionic conductivity, superior electrolyte absorption capability, and tensible deformation. The PAA electrolyte is fragile and facile to rapid dissolution upon exposure to moisture, owing to its high water content and linear chain structure [99,100,101]. Introducing other components into PAA can improve its stability [102,103,104]. For instance, the porous PVA/PAA composite nanofibers were fabricated via an electrospinning process [105]. The internally connected porous structure can be generated by immersed PAA in a 6 M KOH electrolyte, thus obtaining an ionic conductivity of 235.7 mS cm^−1^. The PVA acts as the main body and packaging material, which ensures good stability and high flexibility. The composite GPE signified a high rate of performance, high power output, and good lifespan in flexible ZABs. Apart from PVA, the PAA-based electrolyte can also be doped with inorganic salt to improve its water retention capacity. A certain concentration of NaCl in the KOH-PVA hydrogel (NaCl-KOH-PAA) was designed [106]. The GPE can be directly used as an electrolyte without soaking in a KOH solution. The NaCl doping can enhance its water retention capacity without decreasing the ion conductivity (170 mS cm^−1^) and stability. The NaCl-KOH-PAA hydrogel-based flexible ZABs showed a good lifetime at −20 °C. Furthermore, the lifetime can extend from 34 h to 49 h as the increased NaCl concentration (~0–1 M) in the electrolyte.

**Figure 6:**
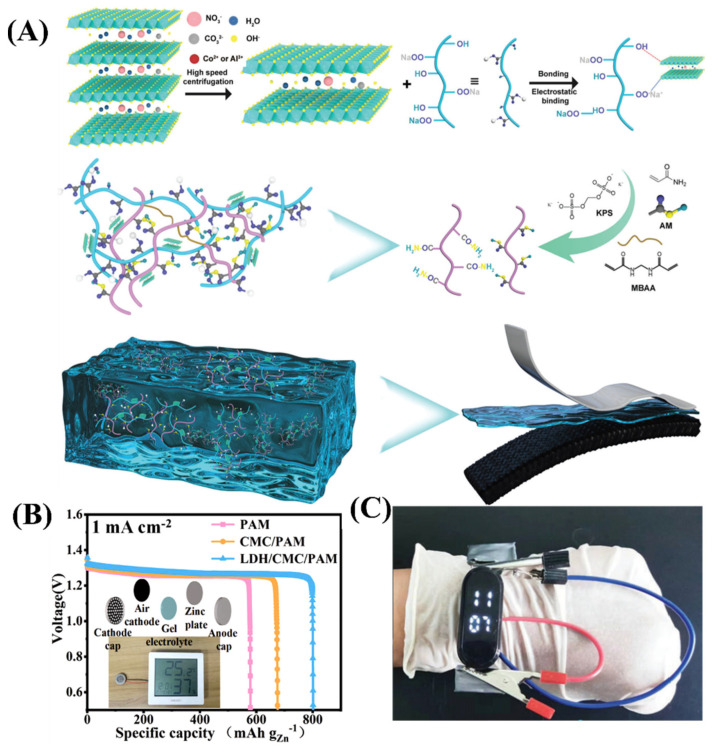
(**A**) Co/Al LDH (Layer double hydroxides) assembled dual network structured nanocellulose/polyacrylamide gel (LDH/CMC/PAM) preparation process diagram. (**B**) Specific capacity profiles at 1 mA cm^−2^. A schematic diagram of a zinc-air button cell and a hygrometer powered by the cell is inset. (**C**) The electronic wristband is assembled with FZAB viewed from the top side. Reprinted with permission from [114], copyright, Wiley VCH.

PAM-based GPEs are widely used in flexible ZABs due to their high ionic conductivity and good interfacial compatibility, but they are limited by mechanical toughness and water retention [107,108,109]. The use of PAM-co-PAA alkaline gel electrolytes has been reported to possess better water retention capacity, higher ion conductivity, and superior stability than the PVA basic gel electrolyte [110]. To further improve the ionic conductivity and mechanical strength of PAM, some strategies, such as the introduction of additives and the construction of dual network gel, have been developed [111,112,113]. For example, the Co/Al layer double hydroxides (LDH) assembled dual network structured nanocellulose/PAM gel was fabricated via the ion exchange, rapid centrifugation, and cross-linking processes [114] (Figure 6A). The Co/Al LDH filled the pores of PAM to achieve high ionic conductivity and high mechanical strength and diminish the water loss. As a result, the ionic conductivity of 0.145 S cm^−1^ and the lifetime of flexible ZAB over 160 h can be obtained. The button cell used in the specific capacity test could drive a hygrometer (Figure 6B). The smartwatch can be powered by the flexible ZAB for use (Figure 6C). The hydrogen-bond network of PAM can be modified by the addition of dimethyl sulfoxide to mitigate Zn dendrite formation and suppress side reactions, which increases the electrochemical interface stability via modulating the cationic solvation structure [115]. The resultant quasi-solid-state ZABs using an atomically dispersed Co electrocatalyst showed good cycling stability with high-capacity retention at −60 and 60 °C.

### 6.2. Battery Configuration for Flexible ZABs

The battery configurations for flexible ZABs often contain sandwich type, coplanar type and cable/fiber type. In a sandwich-type flexible ZAB, the air electrode, the Zn anode and electrolyte are assembled layer by layer in parallel, wherein the electrolyte sandwiched in the middle by the electrodes [116,117]. The stretchability of sandwich-type ZAB was extensively explored.

**Figure 7:**
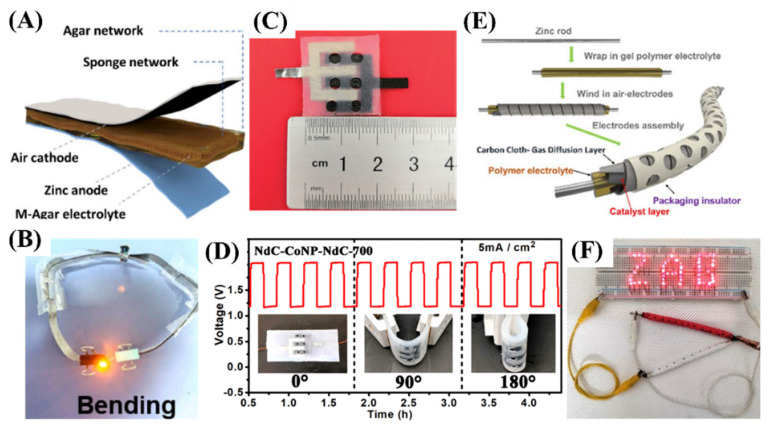
(**A**) Schematic diagram of flexible ZAB based on Agar electrolyte. (**B**) Practical application test of ZAB under bending conditions. Reprinted with permission from [118], copyright, Elsevier. (**C**) An optical photograph of assembled single on-chip all-solid-state ZAB device. (**D**) Galvanostatic discharge/charge cycling curves of on-chip ZAB under bending deformation in different angles (as depicted by the inset images). Reprinted with permission from [122], copyright, The Royal Society of Chemistry. (**E**) Schematic of the assembled rechargeable cable-type all-solid-state ZAB. (**F**) the cable-type all-solid-state ZAB supplies power to the LEDs. Reprinted with permission from [127], copyright, The Royal Society of Chemistry.

For instance, the Agar-based solid-state electrolyte was prepared [118]. As shown in Figure 7A, the Zn foil, Agar electrolyte, and air electrode were assembled into flexible ZAB with a sandwich structure layer-by-layer. The Mn-Co-Fe@CNT supported on nickel foam served as the catalyst. The stability of Agar-based ZAB under various bending states (0 to 180°) was measured, showing good flexibility (Figure 7B). Two pieces of ZABs can stably power the diode by bending two ZABs to 90°. In another example, the starch-based superabsorbent hydrogel polymer electrolyte functionalized with negatively charged carboxyl groups was designed and used in ZABs [119]. The electrolyte possessed high ionic conductivity, electrolyte absorption and retention capabilities, and strong alkaline resistance. The ZABs with a sandwich-type battery configuration showed a long cycle life of 300 h. The stable cycling profile was demonstrated under various deformations, including flatting, bending, and folding states.

Compared with the sandwich-type ZAB, both the air electrode and Zn anode of the coplanar-type ZAB are loaded on the same side of the gel electrolyte [120,121]. It offers ZABs better mechanical flexibility, electrochemical stability, and coplanar integrability. For example, the on-chip all-solid-state ZABs were developed using a carbon cloth coated with N-doped carbon/cobalt-nanoparticle/N-doped-carbon as the air cathode, an interdigital Zn foil as the metal anode and the polyacrylamide-co-polyacrylic/6 M KOH alkaline gel as the solid-state electrolyte [122] (Figure 7C). The ZABs presented good flexibility and coplanar integration capability, offering a specific capacity of 771 mA h g^−1^ and rechargeability of 150 cycles per 50 h (Figure 7D). The coplanar configuration is favorable for the reduction in ionic diffusion resistance, thus improving the performance of ZABs. In addition, it also facilitates the miniaturization and in-planar integration of the device and ensures compatibility with microelectronics.

The cable/fiber-type ZAB presents a 1D coaxial cylinder structure, wherein the Zn anode is coiled to form a spiral structure and is coated by the hydrogel electrolyte. It demonstrates superior flexibility and higher volumetric energy density than sandwich-type batteries [123,124,125,126]. For example, the cable-type flexible ZAB was constructed with a Zn rod as the anode, the Co_2_P/Co encapsulated with nitrogen-doped carbon nanotubes and N,P co-doped graphene sheets (Co_2_P/Co@N-CNT/NPG) as the air cathode and a PVA hydrogel film with 6 M KOH as electrolyte [127]. A Zn rod was placed in a cylindrical template, and then the polymer electrolyte solution was poured into the template. After the hydrogel was cooled in a freezer, the air cathode was wound on it and packaged with a plastic tube (Figure 7E). The as-constructed ZABs exhibited a high peak power density of 145 mW cm^−2^, a capacity of 781 mA h g^−1^, and good stability for 800 h. Two ZABs can supply power for a series of light-emitting diodes (LEDs) (Figure 7F).

## 7. Conclusions

The continuous development of electric vehicles and electronic products has put forward higher requirements for low-cost, high energy density, and high-safety rechargeable air battery devices. Due to their intrinsic virtues, rechargeable ZABs demonstrate great potential for becoming the next-generation energy storage devices. In this review, we have introduced the strategies proposed so far to pursue high-efficiency ZABs and surveyed the recent progress of ZABs from the perspectives of electrocatalysts, electrode chemistry, electrode engineering, aqueous electrolytes, and battery configuration. However, even with this progress, the practical applications of ZABs are still in the infancy stage, and several challenges and technical hurdles exist.

First, high-efficiency bifunctional oxygen electrocatalysts of air cathodes are prerequisites for ZABs. Among various electrocatalysts, non-noble metal electrocatalysts such as hybrid metal catalysts and carbon-supported metal catalysts can overcome the disadvantages of single functionality, poor stability, high cost, and scarce resources of noble metal catalysts, which is worthy of further exploration. Non-noble metal electrocatalysts with hierarchically porous structures and abundant active sites are essential for obtaining optimized charge transport and high-rate energy storage. In this regard, the mechanisms for complex ORR/OER processes need in-depth understanding via theoretical calculations and in situ characterization techniques.

Second, replacing the OER with the organic oxidation reactions could decrease the charging voltage and increase the overall energy conversion efficiency of ZABs. Novel organic oxidation reactions with lower theoretical thermodynamic potential than OER should be further investigated. The electrocatalytic kinetic and/or mechanism studies for related electrochemical reactions such as UOR should be further explored. From the perspective of electrode engineering, the free-standing electrode can be directly used as the air cathode in ZABs without the binder, thus reducing the cost and improving the battery stability. More novel and facile synthesis methods of free-standing electrodes and performance evaluation criteria are required to be developed.

Third, for the liquid ZABs, the commonly used alkaline and mildly acidic electrolytes with superior ionic conductivity often lead to a low stability window and hydrogen evolution. Novel additives for electrolytes should be introduced to overcome the issue. The development of neutral/near-neutral electrolytes can alleviate dendrite growth, hydrogen evolution, electrode corrosion, and passivation of Zn anode in the conventional alkaline electrolyte. Innovative electrolytes and corresponding novel reaction mechanisms are highly desirable and remain a great challenge. In addition, flexible ZABs have attracted a lot of attention due to their high specific energy density, excellent stability, and high safety. The configuration of flexible ZABs requires further optimization to meet the needs of practical applications.

In summary, the construction of high-efficiency ZABs needs the rational design of electrocatalysts, electrode chemistry, electrode engineering, electrolyte, and battery configuration. Till to now, certain constraints exist on the development and combination of each component, which originate from the key device requirements such as efficiency, stability, and scalability. The ultimate target of scientific research is practical application. Without a doubt, the continuous optimization of ZABs from the above aspects could accelerate the development of ZABs with highly efficient, low cost, good safety, and superior stability.
